# Social Network Analysis of e-Cigarette–Related Social Media Influencers on Twitter/X: Observational Study

**DOI:** 10.2196/53666

**Published:** 2024-04-01

**Authors:** Runtao Zhou, Zidian Xie, Qihang Tang, Dongmei Li

**Affiliations:** 1 Goergen Institute for Data Science University of Rochester Rochester, NY United States; 2 Department of Clinical and Translational Research University of Rochester Medical Center Rochester, NY United States

**Keywords:** social network, social media, influencer, electronic cigarettes, e-cigarette, vaping, vape, Twitter, observational study, aerosol, consumer, influencers, social network analysis, antivaping, campaigns

## Abstract

**Background:**

An e-cigarette uses a battery to heat a liquid that generates an aerosol for consumers to inhale. e-Cigarette use (vaping) has been associated with respiratory disease, cardiovascular disease, and cognitive functions. Recently, vaping has become increasingly popular, especially among youth and young adults.

**Objective:**

The aim of this study was to understand the social networks of Twitter (now rebranded as X) influencers related to e-cigarettes through social network analysis.

**Methods:**

Through the Twitter streaming application programming interface, we identified 3,617,766 unique Twitter accounts posting e-cigarette–related tweets from May 3, 2021, to June 10, 2022. Among these, we identified 33 e-cigarette influencers. The followers of these influencers were grouped according to whether or not they post about e-cigarettes themselves; specifically, the former group was defined as having posted at least five e-cigarette–related tweets in the past year, whereas the latter group was defined as followers that had not posted any e-cigarette–related tweets in the past 3 years. We randomly sampled 100 user accounts among each group of e-cigarette influencer followers and created corresponding social networks for each e-cigarette influencer. We compared various network measures (eg, clustering coefficient) between the networks of the two follower groups.

**Results:**

Major topics from e-cigarette–related tweets posted by the 33 e-cigarette influencers included advocating against vaping policy (48.0%), vaping as a method to quit smoking (28.0%), and vaping product promotion (24.0%). The follower networks of these 33 influencers showed more connections for those who also post about e-cigarettes than for followers who do not post about e-cigarettes, with significantly higher clustering coefficients for the former group (0.398 vs 0.098; *P*=.005). Further, networks of followers who post about e-cigarettes exhibited substantially more incoming and outgoing connections than those of followers who do not post about e-cigarettes, with significantly higher in-degree (0.273 vs 0.084; *P*=.02), closeness (0.452 vs 0.137; *P*=.04), betweenness (0.036 vs 0.008; *P*=.001), and out-of-degree (0.097 vs 0.014; *P*=.02) centrality values. The followers who post about e-cigarettes also had a significantly (*P*<.001) higher number of followers (n=322) than that of followers who do not post about e-cigarettes (n=201). The number of tweets in the networks of followers who post about e-cigarettes was significantly higher than that in the networks of followers who do not post about e-cigarettes (93 vs 43; *P*<.001). Two major topics discussed in the networks of followers who post about e-cigarettes included promoting e-cigarette products or vaping activity (55.7%) and vaping being a help for smoking cessation and harm reduction (44.3%).

**Conclusions:**

Followers of e-cigarette influencers who also post about e-cigarettes have more closely connected networks than those of followers who do not themselves post about e-cigarettes. These findings provide a potentially practical intervention approach for future antivaping campaigns.

## Introduction

e-Cigarettes, also known as “e-cigs,” “vapes,” “e-hookahs,” “vape pens,” and “electronic nicotine delivery systems,” heat up a liquid containing propylene glycol, vegetable glycerin, flavors, and frequently nicotine via a battery to produce an aerosol for users to inhale [1,2]. In 2018, 4.5% of respondents sampled in the United States self-reported being current e-cigarette users [3]. Based on the annual national youth tobacco survey from the US Centers for Disease Control and Prevention (CDC) in 2023, e-cigarette is the most popular tobacco product among youth, with 10.0% of high school students and 4.6% of middle school students reported as e-cigarette users [4]. While the CDC suggests that e-cigarettes might be less harmful than regular cigarettes, they have also warned that e-cigarettes are not harmless [5]. One study showed that e-cigarette usage has health effects on seven specific organ systems along with other negative effects to the human body [6]. Another study showed that during the vaporizing process of the e-cigarette liquid, formaldehyde-containing compounds appeared to be formed, and the US National Cancer Institute suggested that formaldehyde is one of the cancer-causing substances in e-cigarettes [7]. Many studies have shown that e-cigarette use is significantly associated with various health risks, including respiratory diseases (such as asthma, chronic obstructive pulmonary disease, and wheezing), cardiovascular diseases, and cognitive defects [8-13].

Given the popularity (especially among youth) and the adverse health effects of e-cigarettes, it is critical to understand how e-cigarettes have become so popular and, more importantly, how to educate the public, especially youth, about their health risks.

The tobacco industry has recognized the power of social media. Accordingly, the industry actively promotes tobacco products or tobacco product use, and mounts opposition to regulatory policies on social media platforms such as Twitter (rebranded as X in July 2023), Facebook, Instagram, and YouTube by hiring social media influencers [14-19]. Conversely, to prevent and reduce tobacco use, the US Food and Drug Administration (FDA) launched “The Real Cost” campaign in 2014 to disseminate antitobacco messages on social media [20]. Several studies have shown that exposure to e-cigarette advertising and promoting messages (eg, on social media) can increase interest in e-cigarettes and the likelihood of initiating e-cigarette use [21-29]. For example, McCausland et al [30] showed that conversations about e-cigarettes on Twitter in Australia generally encourage e-cigarette use. Huang et al [14] found that Twitter appears to be a popular marketing platform for e-cigarette products, with most e-cigarette–related tweets being commercial tweets. Since e-cigarette companies cannot directly market their vaping products online, they typically pay social media influencers (users who have a large number of social media followers) to promote their products [31]. While there are no official definitions or hard thresholds for a social media influencer, most previous studies have defined influencers as social media users who have a large number of followers, remain relevantly active, and gain financial rewards from their activities on social media platforms [32-34]. On Instagram, e-cigarette influencers often promote different brands of e-cigarette products [32,35]. Considering the large number of followers, the posts from e-cigarette influencers can reach many social media users. Studies have shown that higher exposure to e-cigarette–promoting content was significantly associated with e-cigarette use [27,36,37]. In addition, social media influencers posted some misinformation about e-cigarettes (eg, e-cigarettes are harmless) on YouTube [38]. To the best of our knowledge, there has been no study focused on understanding the nature of e-cigarette influencers on Twitter.

Most studies in this field have focused on the association between social media exposure and e-cigarette perception and initiation, and although some of the data analyzed may have covered e-cigarette influencers, the social networks among e-cigarette–related social media influencers and general users are relatively less studied [14,30]. On social media, topical influencers play a vital role in information diffusion within the social network [39-41]. One study tried to reveal the interaction between social media influencers and e-cigarette brands on Instagram using social network analysis [32]. However, the social networks of e-cigarette influencers on social media remain elusive, even though they are critical for understanding how the information is disseminated.

Clustering analysis can provide valuable insights into the structure, behavior, and dynamics of social interactions among users within social networks, which can facilitate the exploration of commonalities within each cluster and potential effective targeted marketing [42]. As one of the fundamental measures, the clustering coefficient can help us understand the network’s architecture by measuring how well-connected users are [43], which can reflect information sharing.

In this study, we aimed to identify e-cigarette–related influencers on Twitter and measure the social network features (eg, the clustering coefficient) between different types of e-cigarette influencers. Although e-cigarette influencers also post tweets unrelated to e-cigarettes, it is of great interest to compare the clustering measures for followers who have posted e-cigarette–related tweets and those who have not, which can help us understand how followers of e-cigarette–related posts are connected. Therefore, we compared the clustering measures between the networks of these two types of followers of e-cigarette influencers. Our findings will provide a more compressive understanding of the differences between different types of e-cigarette influencers, discern the types of prevailing discussions within each influencer’s network, reveal the social network structure of e-cigarette–related influencers on Twitter, and provide a potentially novel intervention strategy for effectively disseminating health-related information for future antivaping campaigns.

## Methods

### Data Collection

Twitter data related to e-cigarettes were collected through the Twitter streaming application programming interface using e-cigarette–related keywords such as “e-cigarette” and “vaping” [44]. From November 19, 2019, to June 10, 2022, we collected 15,787,239 English tweets related to e-cigarettes. For each tweet, we collected as much information as possible, such as tweet ID, created date, text, retweets, favorites, user ID, user name, user tweets, followers count, friends count, and hashtags. In this data set, we identified 3,617,766 unique Twitter user accounts.

### Identifying e-Cigarette Influencers

To identify e-cigarette influencers on Twitter, we defined the following selection criteria based on previous studies [32-34]: (1) social media influencers must have at least 1000 followers, (2) the follower and following ratio for social media influencers must be at least 2:1, (3) social media e-cigarette influencers have posted at least 10 e-cigarette–related posts within the past year, and (4) social media e-cigarette influencers should have posted at least one promotional tweet related to e-cigarettes within the past year.

We iteratively checked the users and tweets in our database against the above criteria and filtered out the Twitter accounts that did not match the criteria. We used keyword matching to determine the promotional tweets related to e-cigarettes [44]. Toward this end, we created a similar list that contained the most common keywords related to promotional tweets and we compared all of the tweets of the candidate influencers against the keywords in this list. If any words in the tweets matched any of the keywords in the list, we categorized the tweets as promotional tweets. At the end of the process, we collected 33 accounts that completely satisfied the above requirements and were not from the e-cigarette industry, vape shops, or retailers.

Based on the tweet content related to e-cigarettes, through a group discussion of all four authors (RZ, ZX, QT, and DL), we split the 33 identified influencers into three broad influencer categories. The first category is “vape advocates,” who provide educational content about e-cigarettes and aim to persuade smokers to switch from traditional cigarettes to e-cigarettes owing to multiple health benefits. The second category is “vape reviewers,” who tweet about their reviews of certain e-cigarette products/devices. The third category is “other,” encompassing Twitter accounts owned by other vaping-related groups that are not affiliated with any vaping brands, such as vaper social network platforms or even vape expos. The tweets from all 33 influencers were carefully examined, and the influencers’ categories were assigned by the coders based on the contents of their tweets and account information.

### Social Network Analysis

Since we were particularly interested in e-cigarette–related influencer social networks, we decided to group the followers of each e-cigarette influencer into those posting about e-cigarettes and those not posting about e-cigarettes, defined as those who posted at least five e-cigarette–related tweets in the past year and those who had not posted any e-cigarette–related tweets in the past 3 years, respectively. To maximize the differences between these two follower groups, we did not include followers of e-cigarette influencers who posted 1-4 e-cigarette–related tweets in the analysis. Considering the large number of followers for each influencer, we randomly sampled 100 followers who post about e-cigarettes and 100 followers who do not post about e-cigarettes from each of the 33 e-cigarette influencers for further social network analysis.

For each e-cigarette influencer, we constructed two social networks for each follower type. We then compared key measures of these networks, including the average clustering coefficient, in-degree centrality, out-degree centrality, closeness centrality, and betweenness centrality, for each social network. The clustering coefficient of a network measures the degree to which nodes in the networks tend to cluster or form groups; a higher clustering coefficient suggests that nodes within the network are more likely to be connected, which can impact the overall structure and function of the network. In-degree centrality measures the number of incoming edges or connections of a node in a directed network, representing the popularity, influence, or attention received by one node from other nodes in the network. Similar to degree centrality, out-degree centrality focuses on the number of outgoing edges or connections from a node in a directed network, representing the extent to which a node reaches out to other nodes and influences them. Betweenness centrality measures the extent to which a node lies on the shortest paths between other pairs of nodes in a network, whereas closeness centrality measures how close a node is to all other nodes in a network. Both betweenness centrality and closeness centrality have significant control over the network’s communication dynamics and are crucial for maintaining efficient information flow.

Two-sample *t* tests and generalized linear models were used to compare the social network metrics between followers who do and do not post about e-cigarettes using R statistical analysis software (R Core Team, 2017) with the significance level set at 5%.

### Topic Modeling Analysis

We performed topic modeling analysis using the latent Dirichlet allocation (LDA) model on all e-cigarette–related tweets posted by the 33 influencers [45]. Further, we identified 100 unique sampled e-cigarette followers for each of the 33 influencers and collected 3.48 million e-cigarette–related tweets posted by these followers in the past 12 months. After standard data cleaning procedures were applied to all tweets, such as removing hashtags, URLs, and emojis [46], we used the LDA model to obtain the most popular topics [45]. We chose the number of topics based on the coherence score and the intertopic distance.

### Ethical Considerations

This study was reviewed and approved by the Research Subjects Review Board of the Office for Human Subject Protection at the University of Rochester (STUDY00006570). This study is a secondary analysis of publicly available Twitter data. All study data have been deidentified before analysis.

## Results

### Identification of e-Cigarette Influencers on Twitter

Between November 19, 2019, and June 10, 2022, we identified 3,617,766 unique Twitter user accounts that posted at least one e-cigarette–related tweet. Among these, 126 Twitter accounts were determined to be e-cigarette influencer candidates based on our criteria. Among them, 33 Twitter accounts were ultimately characterized as e-cigarette influencers, while the remaining 93 were vape shop or company accounts.

Among the 33 e-cigarette influencers, 10 belonged to the category of “vape advocates,” 19 were “vape reviewers,” and four were classified in the “other” category. “Vape advocates” (14,748 followers on average) had more followers than found in the “vape reviewers” (7426 followers on average) and “other” (4683 followers on average) categories. By comparison, “vape reviewers” had a higher percentage of followers that also post about e-cigarettes (1152/7426, 15.5%) than found for the influencer categories “vape advocates” (1357/14,748, 9.2%) or “other” (398/4683, 8.5%). e-Cigarette influencers categorized as “vape reviewers” and “vape advocates” had almost the same proportion of e-cigarette–related tweets at 53.1% (8205/15,454) and 53.2% (8,668/16,295), respectively. Influencers from the “other” category had a slightly higher proportion of e-cigarette–related tweets at 55.7% (3035/5449).

Further examination of all 19,908 e-cigarette–related tweets posted by the 33 e-cigarette influencers identified three major topics discussed (Table 1). The most popular topic was “advocating against vaping policy,” followed by “vaping helps to quit smoking” and “vaping product promotion.”

**Table 1:** Major topics discussed by the 33 e-cigarette influencers on Twitter from November 19, 2019, to June 10, 2022.

Topics	Tweets (N=19,908), n (%)	Top 10 keywords	Example tweets
Advocating against vaping policy	9556 (48)	People, Products, Don't, Ban, THC, Health, Harm, Public, Know	“Years and years from now. When cigarette smoking is finally eliminated. When no one is dying from COPD. You will realize that you wanted JUUL punished for eliminating youth smoking and #vaping is absolutely a public health benefit.” “The number of ‘Vaping is illegal in my country but it helped me quit smoking and now I can't get vape liquids so I had to buy cigarettes’ comments I get on YouTube is far... far too high. Banning #vaping is anti public health. Discouraging people who WANT to quit is shameful.”
Vaping helps to quit smoking	5774 (28)	Quit, Wevapewevote, Worldvapeday, Smokers, Health, Quitlying, Study, Like, Blackmarketthc, Today	“#vaping will change the world and flavors will help it. I do believe this. Everything improves when you quit combustion.” “Ahh yes, the Science. Smokers who switch to vaping may take up healthier routines, new UW study shows, Smokers accidently quit with vaping”
Vaping product promotion	4778 (24)	Vapefam, Vapelife, New, Eliquid, Vapecommunity, Follow, Vapepen, Online, Dabs, Shops	“Arez 120 mod for a thorough test drive!” “Ecigclick Vape Awards 2020 - Final Polls Now Open - Also, be sure to check out our awards giveaway! Biggest prize offering of the year!”

### e-Cigarette Influencer Social Networks

**Figure 1:**
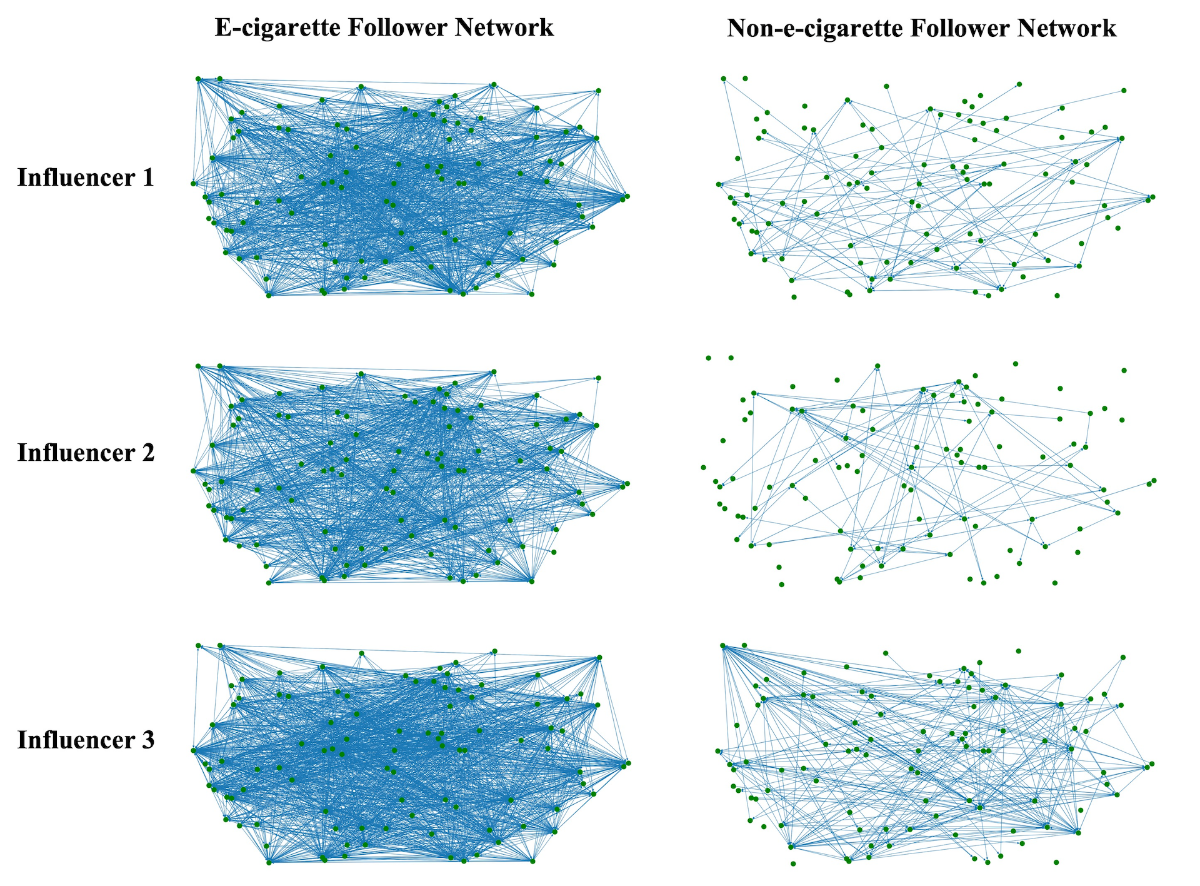
Representative social networks of the followers of e-cigarette influencers who themselves did (e-cigarette follower) or did not (non-e-cigarette follower) post about e-cigarettes on Twitter from November 19, 2019, to June 10, 2022.

The followers of the 33 identified e-cigarette Twitter influencers were grouped into those who previously did or did not post e-cigarette–related tweets. Considering the large number of followers (range 1097-49,215) for each influencer, we randomly selected 100 followers from each group (with or without e-cigarette–related posts of their own) for each influencer to create individual social networks based on their interactions (how they follow each other). To better illustrate the density differences between the social networks of the two types of followers, we randomly selected three e-cigarette influencers to demonstrate their sampled followers’ social networks. As shown in Figure 1, for any of the three representative influencers, the social network of followers who post about e-cigarettes had more connections (edges) than that of followers who do not post about e-cigarettes.

As shown in Table 2, the average clustering coefficient for the networks of followers who post about e-cigarettes was significantly higher than that of the networks of followers who do not post about e-cigarettes. Therefore, the networks of followers posting about e-cigarettes have more connections than those of followers not posting about e-cigarettes for these 33 e-cigarette influencers. In addition, other social network metrics were significantly higher for the followers who post about e-cigarettes compared to those of followers who do not post about e-cigarettes, including in-degree centrality, closeness centrality, betweenness centrality, and out-of-degree centrality (Table 2). Therefore, the social networks of followers of e-cigarette influencers who also post about e-cigarettes have much more incoming and outgoing connections than those of the social networks of followers who do not post about e-cigarettes.

**Table 2:** Comparison of network measures between the networks of followers of e-cigarette influencers who did and did not post about e-cigarettes on Twitter from November 19, 2019, to June 10, 2022.

Social network measures	Followers who post about e-cigarettes	Followers who do not post about e-cigarettes	*P* value
Clustering coefficients	0.398	0.098	.005
In-degree centrality	0.273	0.084	.02
Closeness centrality	0.452	0.137	.04
Betweenness centrality	0.036	0.008	.001
Out-of-degree centrality	0.097	0.014	.02

In addition, we compared the number of followers and their tweets in the social networks of the two e-cigarette influencer follower groups. The average number of follower accounts for followers who post about e-cigarettes was significantly higher than that for followers who do not post about e-cigarettes (322 vs 201; *P*<.001; see Multimedia Appendix 1). Furthermore, the average number of tweets posted in the past 36 months by followers who post about e-cigarettes was higher than that of followers who do not post about e-cigarettes (93 vs 43; *P*<.001; see Multimedia Appendix 2). Considering the higher number of followers and more tweets in the networks of followers who post about e-cigarettes, we decided to fit a generalized linear model to account for these differences. After controlling for the number of followers and tweets, compared to that of the networks of followers who do not post about e-cigarettes, the clustering coefficient of the network of followers who post about e-cigarettes was significantly higher (β=.26, *P*<.001).

Social network measures of the followers who post about e-cigarettes differed between vape advocate and vape reviewer influencers (Table 3). The followers of vape reviewers had higher clustering coefficients than those of vape advocates, which indicates that the e-cigarette–posting followers of vape reviewers have tighter connections than those of vape advocates. However, the network of followers of vape advocates had significantly higher in-degree centrality and closeness centrality compared to those of the networks of followers of vape reviewers. This finding indicates that, on average, the e-cigarette followers of vape advocates have better information flow between each other and a greater amount of incoming information compared to those of followers of vape reviewers. We did not observe a significant difference in the betweenness centrality and out-of-degree centrality between the followers of vape reviewers and vape advocates.

**Table 3:** Social network measures of follower networks of vape advocate and vape reviewer influencers on Twitter from November 19, 2019, to June 10, 2022.

Social network measures	Vape reviewers	Vape advocates	*P* value
Clustering coefficient	0.428	0.335	.02
In-degree centrality	0.061	0.193	.007
Closeness centrality	0.392	0.459	.008
Betweenness centrality	0.007	0.019	.15
Out-of-degree centrality	0.006	0.004	.52

### Topics Discussed in e-Cigarette Social Networks

Since we found that the followers who post about e-cigarettes were more likely to follow each other than the followers who do not post about e-cigarettes for the 33 e-cigarettes influencers, we further wanted to understand the types of information that these followers were sharing about e-cigarettes. As shown in Table 4, we identified two major topics from e-cigarette–related tweets posted on e-cigarette social networks. The relatively more popular topic was promoting e-cigarette products or vaping activity (74,572/133,881, 55.7%), followed by discussing the ability of vaping to help with smoking cessation and harm reduction (59,309/133,881, 44.3%).

**Table 4:** Major e-cigarette–related topics discussed by followers of 33 e-cigarette influencers.

Topics	Tweets (N=133,881), n (%)	Top 10 keywords	Example tweets
Vaping helping with smoking cessation and harm reduction	59,309 (44.3)	Vapefam, New, Ban, People, Don’t, Like, Know, Need, Time, News	“mother to mother, i’d like to know that vaping is important to us because it helped us stop smoking. Our kids need us to be healthy and able to watch them grow up. My beautiful family deserves a mom who doesn’t smoke.” “only 1 in 5 youth report flavors as a reason they vape. Why don’t you spend your time on policies that will actually be effective rather than trying to ruin the most successful form of tobacco harm reduction to date?” “over two thirds of vapers (68%) said they never thought they would quit smoking until #vaping came along - study of 2000 smokers. It's never too late to make the switch.”
Promoting e-cigarette products or vaping activity	74,572 (55.7)	Products, People, Smokers, Quit, Ban, Adults, Like, Youth, Market, Ecigs	“Have you tried the range with cold shots yet? we recommend that you do! digi vape range is available for wholesale, no moq, further enquiries to shane” “go to our Facebook page and join in the competition. ‘Let's play a game, can you spot our new disposable vapes? try your luck’” “starting my day with the beautiful vintage apple from vapour_distillery check out the fam cloud_legion”

## Discussion

### Principal Findings

In this study, with the identification of 33 influencers related to e-cigarettes on Twitter, we investigated the social network differences between followers of these influencers who do and do not post about e-cigarettes on Twitter. We showed that the social networks of followers who post about e-cigarettes have significantly more connections and higher density than those of followers who do not post about e-cigarettes, as indicated by the higher network clustering coefficient. The social networks of followers who post about e-cigarettes also had higher closeness centrality and betweenness centrality than those of followers who do not post about e-cigarettes, which suggests that the social networks of the former group are more efficient in terms of information dissemination, coordination, and influence. There were two main topics shared in the social networks of the followers who post about e-cigarettes, including vaping helping with smoking cessation and harm reduction and promoting e-cigarette products and vaping activity.

### Comparison With Previous Research

With thousands or even millions of followers, social media influencers can have a significant influence on their followers, especially with respect to behavior changes. Therefore, it is crucial to understand how these influencers might engage with their followers and how they influence their followers through social network analysis. First, it is important to understand who these influencers are. A previous study investigated the influence of e-cigarette influencers on their followers on Instagram, showing that only 2 of 55 e-cigarette influencers used the word “influencer” in their descriptions, instead opting to use words such as “public figure,” “brand ambassador,” “promoter,” “video creator,” “artist,” “photographer,” “blogger,” “model,” or “fitness lover” to describe themselves [32]. In this study, we characterized e-cigarette influencers into different categories, including vape reviewers, vape advocates, and others. We did not observe any obvious difference in the hashtags used in their e-cigarette–related tweets, and most of them were related to vaping (Multimedia Appendix 3). Our topic modeling results of the tweets posted by the 33 influencers showed they were advocating against vaping policy, supporting vaping to help smoking cessation, and promoting vaping products on Twitter. In addition, based on the social network measures, we showed that influencers that advocate for vaping can spread their information to more general Twitter users, as indicated by high in-degree and closeness centrality values. However, influencers that review certain e-cigarette products attract more followers who post about e-cigarettes themselves, as indicated by a high clustering coefficient. Together, these results show that the vape reviewers’ followers tend to have more connections with each other, while the vape advocates’ followers tend to have more efficient information spread among each other.

To understand how social networks might be associated with user behavior, we grouped the influencers’ followers into those who post and do not post about e-cigarettes. Our results showed that the network of followers who also post about e-cigarettes had a higher clustering coefficient than that of the network of followers who do not post about e-cigarettes, suggesting that followers who post about e-cigarettes form a denser network (more connections) than followers who do not post about e-cigarettes. While we do not know if this tight connection among followers who post about e-cigarettes is due to their shared interest in e-cigarettes, the messages about e-cigarettes are much easier to share among these followers. In addition, besides the connection between the e-cigarette influencers and followers who also post about e-cigarettes, we found connections between the e-cigarette influencers themselves. For the 33 identified e-cigarette influencers, the number of followers who were influencers ranged from 0 to 17 (Multimedia Appendix 3). Therefore, users with similar interests tend to form a more closed network or community on social media. A previous study showed that social networks with active posting of alcohol-related messages were significantly associated with more frequent alcohol use [47]. It is plausible to speculate that the denser network for followers who post about e-cigarettes might indicate a greater influence on vaping behaviors, such as being more likely to vape and more frequent vaping.

Recognizing the significance of social networks on social media in disseminating e-cigarette–related messages, tobacco companies and vape shops have used social media influencers to promote their e-cigarette products. A previous study showed that, on average, each e-cigarette influencer on Instagram was sponsored by more than 10 different e-cigarette brands such as Voopotech, Innokin, and Geekvape [32]. Moreover, despite the FDA’s efforts to reduce e-cigarette advertisements targeting adolescents on social media, only 25% of the sampled e-cigarette influencers indicated age restrictions to access their e-cigarette promotional posts on Instagram [48]. These influencers on Instagram are very impactful, and their promotional posts through interconnected social networks can reach tens of thousands of adolescents. While more attention has focused on the influence of social media influencers on promoting e-cigarettes, it is important to understand how these influencers influence their followers through social networks. In this study, we showed that, on average, the followers who post about e-cigarettes have more connections in the sampled 100-node network compared to those of followers who do not post about e-cigarettes. The dense network might help disseminate e-cigarette–related promoting messages to more users in the social network, which can be a potentially effective strategy to effectively communicate health education messages with the public for future digital tobacco education campaigns.

Provaping messages are dominant on social media [49-51]. One study demonstrated that while the FDA’s sponsored antivaping hashtag “TheRealCost” only appeared 50 times a month on Instagram, provaping hashtags such as “e-juice” or “e-liquid” appeared more than 1000 times [52]. Another study showed that despite the FDA’s requirement to add warning labels on e-cigarette advertisements on social media, most e-cigarette promotional images have no warning labels [53]. Our study showed that all e-cigarette–related influencers were promoting vaping as a smoking cessation tool and various vaping products on Twitter/X. Besides posting provaping messages, vaping advocates also posted messages advocating against tobacco regulatory policies, as shown in the major topics discussed by the e-cigarette influencers on Twitter/X. The vaping advocates directly contribute highly negative comments to FDA communication efforts, such as the FDA flavor enforcement policy and the proposed rules on menthol cigarettes [30,54-57]. These antiregulatory posts on social media might have a negative effect on the effectiveness of the FDA regulatory efforts. In addition, we noticed that some tweets from e-cigarette influencers are spreading misinformation about e-cigarettes, such as vaping being harmless, which is consistent with findings from other social media studies [58-60]. The spreading of this misinformation about e-cigarettes on social media, especially within social networks or communities, might undermine public understanding of the health risks associated with e-cigarettes, thereby promoting the initiation or continuation of vaping.

In our study, two topics from the networks of followers who post about e-cigarettes were vaping helping with smoking cessation and harm reduction and promoting e-cigarette products and vaping activity. The most popular hashtag we found in this social network was “WeVapeWeVote” (Multimedia Appendix 3), an organization fighting to protect the rights of adults to access vapor devices and other smokeless alternatives. The Hashtag “Vapefam,” used by someone who views the vaping community as an extended family, has also been mentioned more than 65,000 times in our data set. It is reasonable to speculate that these dominant provaping messages on social media might push back against current efforts of vaping regulation policies (such as the FDA’s flavor enforcement policy) that aim to reduce the current vaping epidemic in youth, which should draw more attention from public health authorities. Further, more educational warning messages about the health risks of e-cigarette use should be encouraged and promoted on social media, which future tobacco education campaigns should consider. In contrast, as shown in Multimedia Appendix 3, the top 10 hashtags in tweets posted by the followers who do not post about e-cigarettes were more likely to be related to health, such as #health and #selfimprovement.

### Limitations

There are several limitations of this study. First, the definition of followers who post about e-cigarettes was based on Twitter user accounts that have posted at least 10 e-cigarette–related tweets within the last 12 months. However, it is uncertain whether these posters are also e-cigarette users. In future studies, we will determine if they are actual e-cigarette users through use of a human-guided deep-learning model. Second, other factors (such as the demographics of Twitter users) might partially influence the average clustering coefficients, which need to be controlled in the future. In this study, we did not investigate the impact of social networks on user behaviors, which can be further measured in the future through a longitudinal study. Finally, the social network is dynamically evolving. Therefore, it is important to monitor the social network longitudinally to study how it evolves and influences user behaviors.

### Conclusion

Through social network analysis, we showed that the social networks of followers of e-cigarette Twitter influencers who themselves also post about e-cigarettes have a denser connection compared to that of followers who do not post about e-cigarettes, which helps us better understand how e-cigarette influencers influence their followers through social networks. More importantly, social networks on social media might provide a novel and effective intervention approach for future health education campaigns to disseminate antivaping messages. For example, considering the dense social networks among e-cigarette social media users, public health advocates can join these social networks by following either the influencer or their followers who post about e-cigarettes, which would enable vaping prevention messages to be disseminated quickly in the networks and to specifically target potential or current e-cigarette users.

## Appendix

Multimedia Appendix 1 Comparison of number of followers in the networks of followers of e-cigarette influencers who post (e-cigarette followers) and do not post (non-e-cigarette followers) about e-cigarettes.

Multimedia Appendix 2 Comparison of the number of tweets posted in the networks of e-cigarette influencer followers who post about e-cigarettes (e-cigarette followers) and do not post about e-cigarettes (non-e-cigarette followers).

Multimedia Appendix 3 Top 10 hashtags observed in e-cigarette-related tweets from e-cigarette influencers (Table S1); Number of followers who themselves are influencers for each e-cigarette influencer (Table S2); Top 10 hashtags in e-cigarette-related tweets from followers (Table S3).

## Abbreviations

CDC: Centers of Disease Control and Prevention
FDA: Food and Drug Administration
LDA: latent Dirichlet allocation

## Data Availability

The Twitter/X data used in the study are publicly available from the Twitter/X website [61]. The Python codes used for data analyses are available upon reasonable request to the corresponding author.
