# Supplementary figures and images for: Social Network Analysis of e-Cigarette–Related Social Media Influencers on Twitter/X: Observational Study
Source: JMIR Form Res. 2024 Apr 1;8:e53666. doi: 10.2196/53666 (PMC11019427; doi:10.2196/53666)

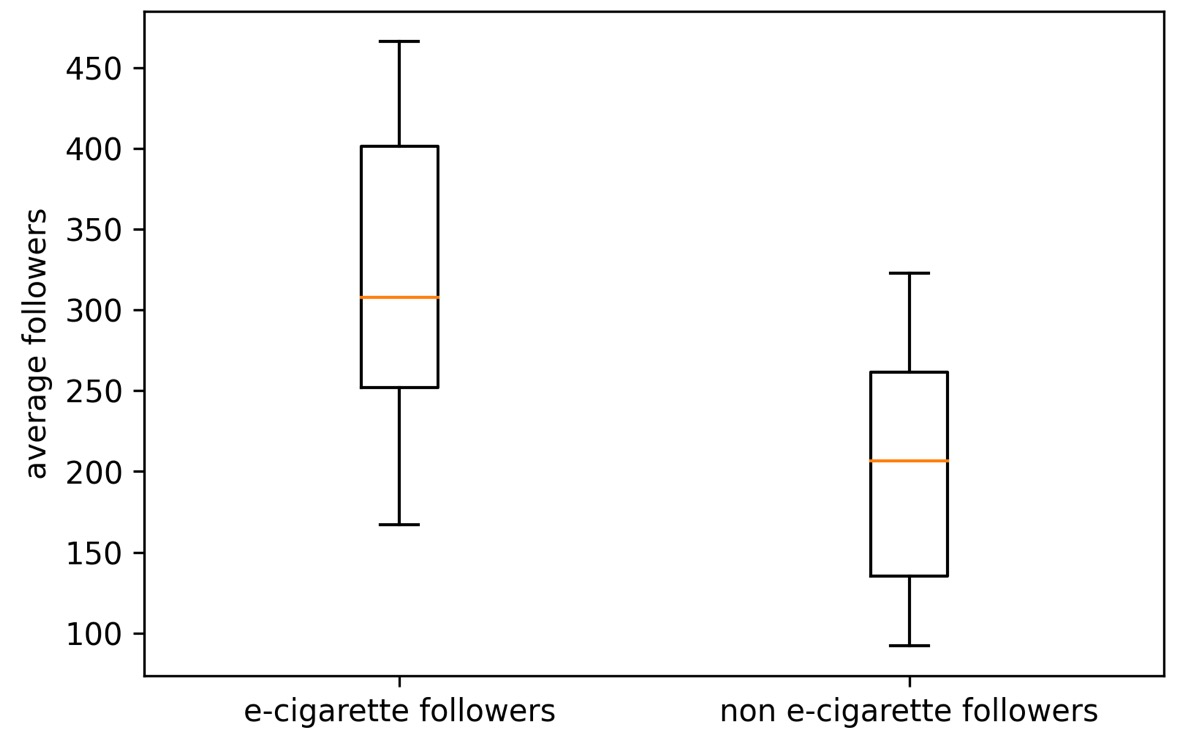

Supplement: Multimedia Appendix 1 [file formative_v8i1e53666_app1.png]

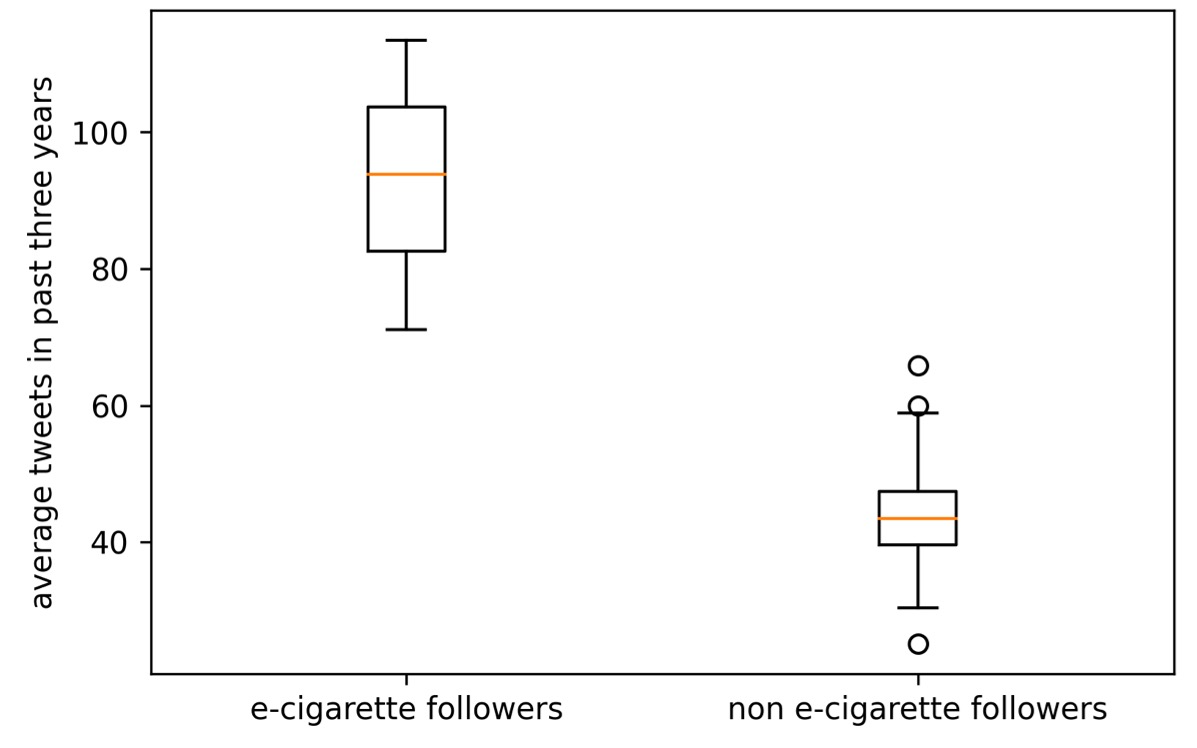

Supplement: Multimedia Appendix 2 [file formative_v8i1e53666_app2.png]
