# Supplementary material for: Social Network Analysis of e-Cigarette–Related Social Media Influencers on Twitter/X: Observational Study
Source: JMIR Form Res. 2024 Apr 1;8:e53666. doi: 10.2196/53666 (PMC11019427; doi:10.2196/53666)
Supplement: Multimedia Appendix 3 [file formative_v8i1e53666_app3.docx]

**Multimedia Appendix 3. Top 10 hashtags observed in e-cigarette-related tweets from e-cigarette influencers (Table S1); Number of followers who themselves are influencers for each e-cigarette influencer (Table S2); Top 10 hashtags in e-cigarette-related tweets from followers (Table S3).**

**Table S1. Top 10 hashtags observed in e-cigarette-related tweets from e-cigarette influencers.**

| **Influencers categories** | | | | | |
| --- | --- | --- | --- | --- | --- |
| **Vape advocates** | | **Vape reviewers** | | **Others** | |
| **Hashtag** | **# Tweets** | **Hashtag** | **# Tweets** | **Hashtag** | **# Tweets** |
| vaping | 136 | vapefam | 539 | vapouround | 41 |
| nicotine | 108 | vapers | 423 | VOXPO | 37 |
| Vaping | 39 | vape | 235 | vaping | 33 |
| harmreduction | 37 | vapelife | 225 | VapouroundAwards2022 | 31 |
| Science4RAJA | 21 | VapeFam | 74 | vapefam | 29 |
| HarmReduction | 18 | vapenation | 27 | VAPELive | 27 |
| vape | 13 | eliquid | 16 | VAPELIVE | 25 |
| science | 13 | vapecommunity | 13 | vape | 23 |
| smoking | 11 | vaporesso | 11 | vapecommunity | 17 |
| vapingsaveslives | 10 | vaping | 10 | vaperexpo | 13 |

**Table S2. Number of followers who themselves are influencers for each e-cigarette influencers.**

| **E-cigarette influencer Id** | **Number of followers who are influencers** | **E-cigarette influencer Id** | **Number of followers who are influencers** |
| --- | --- | --- | --- |
| No. 1 | 17 | No. 18 | 9 |
| No. 2 | 8 | No. 19 | 2 |
| No. 3 | 3 | No. 20 | 7 |
| No. 4 | 12 | No. 21 | 16 |
| No. 5 | 1 | No. 22 | 11 |
| No. 6 | 5 | No. 23 | 4 |
| No. 7 | 10 | No. 24 | 8 |
| No. 8 | 0 | No. 25 | 0 |
| No. 9 | 4 | No. 26 | 1 |
| No. 10 | 6 | No. 27 | 8 |
| No. 11 | 1 | No. 28 | 12 |
| No. 12 | 3 | No. 29 | 7 |
| No. 13 | 9 | No. 30 | 10 |
| No. 14 | 0 | No. 31 | 0 |
| No. 15 | 15 | No. 32 | 4 |
| No. 16 | 6 | No. 33 | 2 |
| No. 17 | 13 |  |  |

**Table S3. Top 10 hashtags in e-cigarette-related tweets from followers.**

| **Follower categories** | | | |
| --- | --- | --- | --- |
| **E-cigarette followers** | | **Non-e-cigarette followers** | |
| **Hashtag** | **# Tweets** | **Hashtag** | **# Tweets** |
| WeVapeWeVote | 86,350 | health | 13,909 |
| vapefam | 65,191 | selfimprovement | 8,571 |
| vape | 51,276 | ukraine | 4,215 |
| vapecommunity | 29,468 | government | 1,862 |
| vapers | 18,693 | omicron | 794 |
| vapelife | 10,351 | family | 517 |
| ecigs | 7,962 | mindfulness | 369 |
| vapenation | 4,557 | publichealth | 142 |
| smoking | 1,529 | elonmusk | 93 |
| health | 926 | love | 68 |
